# Trans-D3: A Novel Hybrid Transformer-Based Actor–Critic Approach for Remaining Useful Life Prediction

**DOI:** 10.3390/s26102949

**Published:** 2026-05-08

**Authors:** Jorge Paredes, Danilo Chavez, Ramiro Isa-Jara, Diego Vargas

**Affiliations:** 1 Departamento de Automatización y Control Industrial, Facultad de Ingeniería Eléctrica y Electrónica, Escuela Politécnica Nacional, Quito 170525, Ecuador; 2 Facultad de Informática y Electrónica, Escuela Superior Politécnica de Chimborazo, Avenida Pedro Vicente Maldonado s/n & Avenida 11 de Noviembre, Riobamba 060104, Ecuador

**Keywords:** predictive maintenance, reinforcement learning, supervised learning, transformer, TD3, remaining useful life

## Abstract

The present article introduces TRANS-D3, an innovative hybrid method that combines the Twin Delayed Deep Deterministic Policy Gradient (TD3) reinforcement learning algorithm with the Transformer architecture for predicting the remaining useful life (RUL). The model utilizes an optimized reward function based on the Linear Quadratic Regulator (LQR) to approach error correction as a dynamic control problem. On the CMAPSS dataset, TRANS-D3 demonstrates a marked advantage, achieving RMSE reductions of 84–90% in baseline situations (FD001) and 23–45% in highly variable contexts (FD003/FD004). Statistical validation demonstrates high reliability, with a coefficient of determination $R^{2}$ of more than 0.93 in each of the subsets; the maximum is 0.9984 in FD001. The 95% confidence intervals for the mean error, ranging from [0.709 to 1.244] in FD001 and from [−1.324 to 1.748] in FD004, also confirm that the framework is a statistically unbiased estimator. In terms of Score, the model reduces penalties by between 80% and 95% compared to advanced architectures such as DAST or STAR, ensuring very stable predictions. These findings present a novel robust optimization paradigm, which is essential for ensuring the safety and reliability of complex industrial systems in the context of Industry 4.0.

## 1. Introduction

The integration of artificial intelligence (AI), the Internet of Things (IoT), and Big Data has enabled a substantial transformation in the digitization of industrial processes under a new paradigm known as “Industry 4.0” [1]. To ensure digital transformation, it is imperative that digital and physical systems be integrated into cyber-physical systems [2], thereby enabling the generation of substantial amounts of data from sensors and actuators. The data can be used to extract useful information from the components of a production line in a factory [3].

Machine learning (ML) is a sub-branch of artificial intelligence that uses specialized algorithms for classification, prediction, pattern detection, and industrial process optimization tasks, thus reducing costs in industrial factories [4]. Furthermore, the preprocessing and analysis of the acquired data can yield valuable insights [5]. Machine learning constitutes a pivotal component of the advancements witnessed in smart factories, as it facilitates the aggregation of data that enables subsequent analysis. This capacity to detect faults in machinery and equipment facilitates the estimation of the remaining useful life (RUL) and health index (HI) of the components in question, thereby reducing maintenance-related expenditures and enhancing operator safety [6,7].

Machine learning algorithms can be classified into three categories: supervised, which utilize labeled data; unsupervised, which employ unlabeled data; and hybrid approaches [8]. Reinforcement learning, which learns from experience and has recently gained significant prominence in the field of machine learning [9], is also a distinct category. The efficacy of these approaches is contingent upon the specific context in which they are employed. Currently, there are methodologies that integrate the aforementioned machine learning algorithms [10].

Predictive maintenance (PdM), also known as condition-based machine (CbM) [11], is an application of machine learning in the context of Industry 4.0. This application allows data to be acquired from sensors, machinery, or systems in order to predict breakdowns within a given time frame or classify faults in order to optimize maintenance tasks [12].

Maintenance activities in the industrial sector can be classified into three categories [13]:

1. Corrective maintenance: performed when a machine or component fails.
2. Preventive maintenance: maintenance tasks are scheduled even if the machinery or component is not malfunctioning.
3. Predictive maintenance: sensor data is used to predict a failure in the machine or components before it occurs, optimizing time and resources.

**Figure 1:**
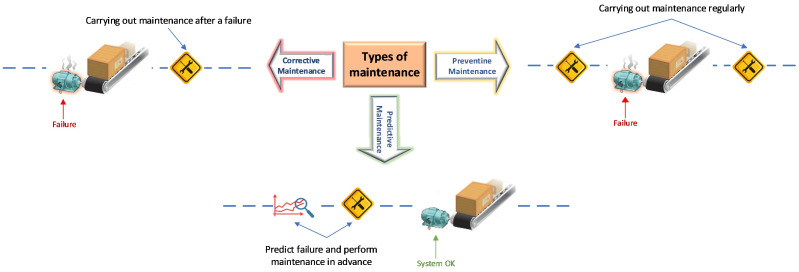
Types of maintenance.

Figure 1 shows a graphical representation of the three categories of maintenance used in the industrial sector.

A primary objective of predictive maintenance is to estimate the RUL of a mechanical or electrical system [14], thereby enabling the prediction of the time interval before failure occurrence, taking into account operating conditions. There are three primary methods for predicting RUL:

1. Physical model-based.
2. Data-driven models.
3. Hybrid models.

Physical model-based methods are constructed using knowledge of the differential equations governing the model and expert knowledge of the analyzed equipment to determine the RUL [15]. When the degradation process is modeled with high precision, very accurate results can be obtained [16]. However, practical implementation can be complex because it requires a substantial understanding of the system, which is sometimes not accessible.

The employment of data-driven models enables the identification of a correlation between the obtained data and the RUL, thereby obviating the necessity of a physical degradation model. These methods are currently widely used due to their great versatility in addressing the complexities inherent in complex systems and equipment [17]. The RUL of lithium-ion batteries can be predicted through the utilization of support vector machines, as evidenced by the findings outlined in [18]. The extraction of salient features in time series has been facilitated by the implementation of deep learning methodologies [19]. Neural networks are algorithms that simulate the human brain and are used in the oil and gas industry to calculate RUL [20]. In scenarios where enhanced precision and accuracy are paramount, the utilization of networks such as Long Short-Term Memory (LSTM) becomes a viable option. These networks possess the capability to analyze time series and eliminate noise, thereby ensuring a more robust and reliable analysis [21]. Furthermore, these networks can be integrated to formulate a Bidirectional LSTM model for RUL prediction [22]. It is important to note that all of these types of approaches are contingent upon the utilization of labeled data. Recent research has demonstrated that Transformers exhibit superior performance in predicting RUL when compared to LSTM and Gated Recurrent Unit (GRU) models, particularly when incorporating learned temporal embeddings [23].

Hybrid models are those that combine different machine learning algorithms. In some models, a semi-supervised approach is employed, integrating both supervised and unsupervised algorithms [24]. In contrast, other models may combine supervised and reinforcement techniques [25]. The RUL of lithium-ion battery banks can be calculated using supervised and reinforcement algorithms, which allows for the incorporation of parameterized policies, thereby reducing inspection times [26]. A decision-making framework can also be proposed to assist operators in recommending maintenance activities [27]. Semi-supervised approaches can be trained with a limited amount of labeled data and subsequently utilized to calculate RUL from unlabeled data [28]. The integration of clustering methodologies with LSTM networks has been demonstrated to enhance the reliability of RUL estimation in lithium battery banks [29]. The most prevalent hybrid models possess the capacity to interact with time series and extract features to determine RUL by combining Convolutional Neural Networks (CNN) and LSTM [30].

The calculation of the RUL can present challenges when employing a single algorithm, irrespective of whether it is supervised, unsupervised, or reinforcement-based. This is due to the potential inadequacy of the algorithm in adequately capturing the intricate relationships between samples [31]. One potential solution to this problem is the utilization of hybrid approaches, which integrate supervised and reinforcement algorithms. These approaches leverage temporal relationships during the training phase, enhancing the precision and accuracy of the RUL.

To provide a clear context for the proposed research within the current state of the art (SOTA), Table 1 offers a structured overview of representative and recent studies on RUL prediction. The following table offers a synopsis of salient aspects of extant approaches, including the year of publication, the datasets or materials employed, the methodologies implemented, and their primary advantages. It also explicitly highlights the research gaps that persist. This comparative analysis underscores that, despite substantial progress in data-driven forecasting, the prevailing methods are predominantly founded on purely supervised learning paradigms and are deficient in an explicit control-oriented mechanism for prediction refinement, particularly under complex and highly variable operating conditions. These observations have led to the development of the proposed framework.

Table 1 offers a systematic synopsis of notable and contemporary studies on RUL prediction, published between 2021 and 2025. As evidenced by the extant literature, contemporary approaches predominantly depend on supervised deep learning architectures (e.g., Transformer variants, attention-based recurrent networks, or hybrid CNN-LSTM models) to enhance temporal feature extraction and predictive accuracy. While these methods have been shown to achieve competitive performance, they generally treat RUL estimation as a static regression problem and lack an explicit mechanism for adaptive error correction or decision-oriented optimization. A closer look at the reviewed studies reveals a glaring omission: the absence of any incorporation of a reinforcement learning or control theory framework. This oversight is particularly salient in the context of dynamic refinement of predictions, a crucial element in response to evolving degradation patterns. This deficiency becomes particularly problematic in highly variable operational environments, where stable and conservative predictions are imperative for safety-critical industrial systems.

To address these limitations, this article proposes a hybrid approach that integrates a supervised algorithm (Transformer) and a reinforcement algorithm (TD3) named TRANS-D3. This framework formulates RUL prediction error correction as a sequential control problem, integrating Transformer-based temporal representation with a TD3 reinforcement learning agent and an LQR-inspired reward function. The utilization of both algorithms enables the maximization of advantages and benefits, thereby ensuring the acquisition of precise and reliable data concerning the RUL of a component or machine, providing greater robustness, stability, and generalization in complex Industry 4.0 scenarios.

The primary contributions of this study are as follows:

1. Innovative hybrid approach, as Trans-D3 integrates a supervised Transformer model with a TD3 reinforcement algorithm, leveraging the advantages of both paradigms to estimate RUL more robustly.
2. An optimized reward function for the reinforcement algorithm, based on the control law of a linear quadratic regulator, which allows for greater stability and control when calculating RUL.
3. Improvement of 35% in RUL prediction accuracy compared to supervised models and 15% over hybrid models, reducing unplanned downtime and maintenance costs by increasing operational reliability.

The following paper is structured as follows: Section 2 presents an exposition of the algorithms that have been utilized. Section 3 delineates the methodological framework employed in the hybrid approach. The results obtained are presented in Section 4. Section 5 discusses the results and potential improvements. Finally, Section 6 contains the conclusions of the research.

## 2. Background

### 2.1. Transformer

In recent years, the Transformer architecture has emerged as one of the most influential contributions to deep learning for sequence modeling. The model’s design, predicated exclusively on attention mechanisms, has enabled it to overcome the inherent limitations of recurrent and convolutional networks in tasks requiring the capture of global dependencies and complex patterns [38]. Transformers have been identified as optimal candidates for engineering problems, including RUL estimation. This is due to their capacity to represent nonlinear relationships between multiple sensors, a critical aspect of the field.

The dot-product scaled attention mechanism constitutes the fundamental mathematical basis of the transformer and is expressed as follows:

(1) $$\mathrm{Attention}\left(Q,K,V\right)=\mathrm{softmax}\;\left(\frac{QK^{⊤}}{\sqrt{d_{k}}}\right)V$$

where *Q*, *K*, *V* and $d_{k}$ are the query, key, value matrices and the key dimensionality, respectively.

This formulation enables the model to discern distributions of importance on the input, a capability that is particularly advantageous in time series with noise or redundancy, such as those originating from aerospace or turbomachinery systems [39].

The multi-head attention (MHA) scheme extends this mechanism by allowing multiple projections:

(2) $$h_{i}=\mathrm{Attention}\left(QW_{i}^{Q},\;KW_{i}^{K},\;VW_{i}^{V}\right)$$

(3) $$\mathrm{MHA}\left(Q,K,V\right)=\mathrm{Concat}\left(h_{1},\ldots ,h_{H}\right)W^{O}$$

where *W* is the trainable weight matrix.

This configuration facilitates the model’s capacity to depict diverse relational configurations within the signal, a pivotal consideration in RUL prediction, where physical degradation manifests across multiple time scales [40].

Positional encoders (PE) address the absence of a temporal mechanism in the original architecture. The proposed sinusoidal formulation is defined as follows:

(4) $$PE_{\left(pos,2i\right)}=\sin\;\left(\frac{pos}{{10,000}^{2i/d_{model}}}\right)$$

(5) $$PE_{\left(pos,2i+1\right)}=\cos\;\left(\frac{pos}{{10,000}^{2i/d_{model}}}\right)$$

where $d_{model}$ is the internal embedding dimension of Transformer

This capability enables the model to concurrently maintain both relative and absolute information about positions in sequence. These encoders have been extensively utilized in studies concerning forecasting and structural degradation [41].

Each sublayer incorporates residual normalization, which is expressed as follows:

(6) $$\mathrm{LayerOutput}=\mathrm{LayerNorm}\;\left(x+\mathrm{Sublayer}(x)\right)$$

This stabilizes the gradient flow, thereby enabling the training of models with extreme depth [42]. This pattern has proven to be particularly relevant in industrial architectures, where datasets tend to be heterogeneous and highly complex [43].

The point-by-point feed-forward (FNN) block is defined as follows:

(7) $$\mathrm{FFN}\left(x\right)=\max\left(0,xW_{1}+b_{1}\right)W_{2}+b_{2}$$

where *x* is the input feature vector and $b_{1}$ and $b_{2}$ are bias vectors.

This component incorporates nonlinear capacity into the model, thereby enabling the representation of highly complex and non-stationary degradation functions [44].

The decoder incorporates a masked attention mechanism that restricts access to future positions. The operational mechanisms of the aforementioned system are delineated as follows:

(8) $$\alpha_{ij}=\left\{\begin{array}{cc} \infty , & j>i,\\ \frac{{\left(QK^{⊤}\right)}_{ij}}{\sqrt{d_{k}}}, & j\leq i \end{array}\right.$$

This procedure is designed to ensure appropriate autoregressive behavior in prediction tasks.

Despite its effectiveness, the computational cost of the original Transformer is quadratic with respect to sequence length, which can hinder its use in real time. However, its capacity to directly capture global interactions has led to significant advancements in industrial forecasting tasks [45].

Alternatives such as Linformer [46] have reduced complexity by approximating the required attention using low-rank projections, while others such as Performer [47] use kernel-based approximations, enabling efficient hardware execution. The implementation of these approaches is increasing in the domain of predictive maintenance, owing to their scalability.

Transformers are a robust, flexible, and mathematically well-founded architecture for modeling complex time series. Their capacity to capture multivariate dynamics and global dependencies establishes them as a promising instrument for precise RUL estimation in industrial systems. The evolution of efficient variants and the incorporation of hybrid techniques continue to expand their potential in forecasting, condition analysis, and intelligent engineering scenarios [48].

**Figure 2:**
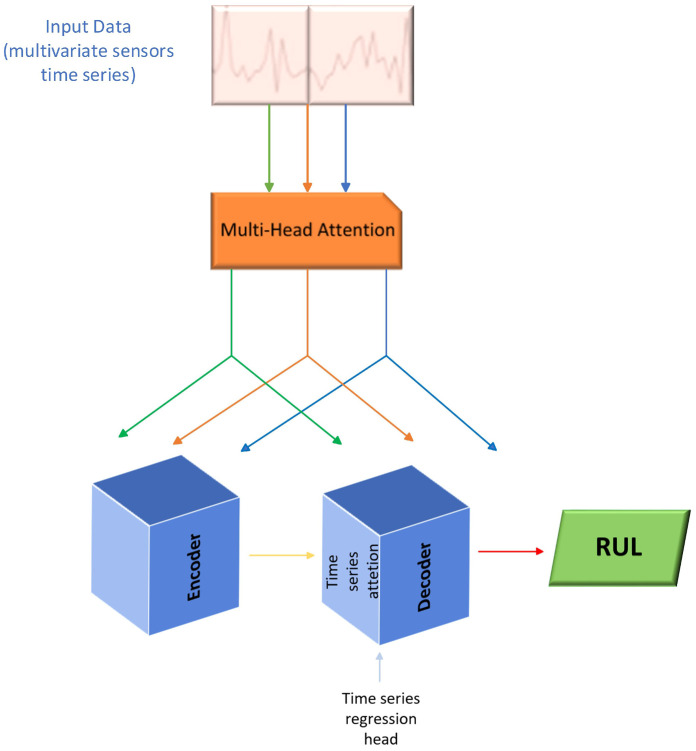
Regression framework using a transformer.

Figure 2 delineates the Transformer network architecture, which has been adapted for time series regression tasks, with the objective of estimating RUL in complex systems. In contradistinction to natural language processing models that operate with word vectors, this structure receives multivariate sensor sequences as input, which are processed through a Multi-Head Attention mechanism. This component is of paramount importance, as it enables the model to discern critical correlations and global dependencies between multiple sensor signals distributed over time, even in the presence of operational noise or data redundancy.

The configuration is based on an encoder–decoder scheme that has been optimized for continuous forecasting. The Encoder extracts a latent representation of the system’s degradation characteristics, while the Decoder incorporates a time series regression head designed to map these abstractions to a single scalar value representing the RUL. This framework facilitates the capture of nonlinear and transient dynamics in component degradation, such as in turbofan engines, thereby providing a robust forecasting tool that overcomes the memory limitations of traditional recurrent networks by processing all available temporal history in parallel and attentively.

### 2.2. TD3

The Twin Delayed Deep Deterministic Policy Gradient (TD3) algorithm is predicated on the continuous reinforcement learning framework with deterministic policies. Its predecessor, Deep Deterministic Policy Gradient (DDPG) [49], learns a deterministic policy $\pi_{\theta }$*(s)* and an action value function $Q_{\phi }$*(s,a)*. The objective is to maximize the expected return.

(9) $$J\left(\theta \right)=E_{s∼D}\left[Q_{\phi }\left(s,\pi_{\theta }\left(s\right)\right)\right]$$

TD3 [50] aims to address the limitations inherent in DDPG, particularly the overestimation of the Q-value. The algorithm introduces three fundamental innovations:

1. Clipped double Q-Learning
2. Target policy smoothing
3. Delayed policy updates

Each of these factors exerts an influence on the manner in which the actor and critic parameters are updated.

The clipped double Q-Learning approach utilizes two independent critics, $Q_{\phi 1}$ and $Q_{\phi 2}$. The critic objective employs the minimum of the two to mitigate overestimation. In the context of a transition $\left(s_{t},a_{t},r_{t},s_{t+1}\right)$, the TD3 objective is defined as follows:

(10) $$y_{t}=r_{t}+\gamma *\min_{i=1,2}Q_{\phi_{i}^{\prime }}\left(s_{t+1},{\tilde{a}}_{t+1}\right)$$

where $\phi_{i}^{\prime }$ i are the parameters of the target networks [51].

The term ${\tilde{a}}_{t+1}$ is the result of target policy smoothing, which adds limited Gaussian noise.

(11) $${\tilde{a}}_{t+1}=\pi_{\theta^{\prime }}\left(s_{t+1}\right)+\epsilon ,\;\epsilon ∼\mathrm{clip}\left(N\left(0,\sigma^{2}\right),-c,c\right)$$

This technique reduces the sensitivity of Q to small variations in action, an idea inspired by gradient stabilizers seen in previous work [52].

The critic is trained by minimizing the quadratic loss.

(12) $$L\left(\phi_{i}\right)=E_{(s_{t},a_{t},r_{t},s_{t+1})∼D}\left[{\left(Q_{\phi_{i}}\left(s_{t},a_{t}\right)-y_{t}\right)}^{2}\right]$$

This design compels both critics to approximate the expected return, albeit always guided by the more conservative value between them.

The third component of TD3 is delayed actor updates. While critics are updated at each iteration, the deterministic policy is only updated every d steps (typically *d* = 2). The actor gradient is derived from the deterministic policy gradient rule [53]:

(13) $$\nabla_{\theta }J\left(\theta \right)=E_{s∼D}\left[\nabla_{a}Q_{\phi_{1}}\left(s,a\right){|}_{a=\pi_{\theta }\left(s\right)}\nabla_{\theta }\pi_{\theta }\left(s\right)\right]$$

Slow policy updating prevents premature propagation of critical estimation errors. Subsequent studies have shown that this improves stability, especially when using deep networks [54]. In addition, the target parameters are updated using a soft update:

(14) $$\phi_{i}^{\prime }\leftarrow \tau \phi_{i}+\left(1-\tau \right)\phi_{i}^{\prime },\;\theta^{\prime }\leftarrow \tau \theta +\left(1-\tau \right)\theta^{\prime }$$

with a typically small τ (0.005).

TD3 has been demonstrated to substantially mitigate the overestimation bias that has been documented since the early function approximation studies in Reinforcement Learning. In contrast to stochastic techniques such as Soft Actor Critic (SAC), TD3 employs a deterministic policy.

(15) $$a_{t}=\pi_{\theta }\left(s_{t}\right)$$

This facilitates more efficient learning in environments where continuous precision is imperative. TD3 employs a similar approach to DDPG, utilizing an experience replay buffer (D). This decoupling of temporal correlation between samples has been demonstrated to enhance performance in complex environments [55].

The employment of dual critics can be interpreted through the lens of uncertainty estimation. Although TD3 does not explicitly model distributions, its mechanism is conceptually similar to bootstrapping-based approaches such as Bootstrapped DQN [56], where multiple heads reduce bias and variance.

TD3 has been demonstrated to attain superior average returns and diminished variance in comparison to DDPG, attributable to its configuration, which renders it more resilient to overfitting in regions characterized by elevated false Q values. Since its publication, TD3 has been extended to applications in robotics, predictive control, and industrial automation. Its influence has also been observed in variants such as MATD3, which incorporate double criticality and smoothed policies [57].

TD3 signifies a substantial advancement in the domain of reinforcement learning. The integration of double criticals, smoothing, and delayed updates has resulted in a robust architecture that has set a standard for predictive control.

**Figure 3:**
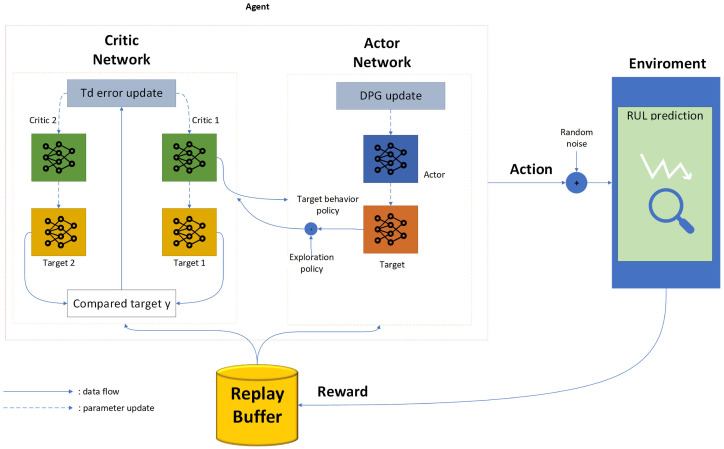
Refining RUL scheme using TD3.

Figure 3 illustrates the architecture of an agent based on the TD3 algorithm. This algorithm is designed as an optimization framework for regression and prediction refinement tasks. In this scheme, the agent operates within a state space, integrating the current estimate and a temporal reference. These are processed through an Actor model to determine the necessary adjustment magnitude. This reinforcement learning structure enables the system to generate predictions and learn a dynamic correction policy that can adapt to deviations observed during the monitoring process.

To ensure training stability and mitigate overestimation errors, the system is equipped with a Twin Critics module. These components evaluate the quality of actions independently, while Target Networks with smoothed updates ensure consistent learning in the long term. A reward loop penalizes accuracy errors, forcing the algorithm to converge towards a policy that maximizes the fidelity of the final prediction. This results in a robust model against noisy signals and nonlinear behaviors in complex systems.

## 3. Methodology

The proposed methodology for RUL prediction is designed to accurately model the temporal evolution of degradation processes in complex industrial systems. In applications such as turbofan engines, the available information consists of multivariate sensor time series characterized by high dimensionality, long-term temporal dependencies, and operational noise. Addressing these challenges requires a modeling framework capable of extracting meaningful degradation representations while preserving both local dynamics and global temporal trends.

To this end, a supervised learning model based on the Transformer architecture is employed as the core component for feature extraction and initial RUL estimation. By leveraging the self-attention mechanism, the Transformer effectively captures long-range dependencies across sensor sequences without the memory constraints inherent to recurrent neural networks. This capability enables the model to learn complex interactions among multiple sensor signals over time, resulting in a preliminary RUL prediction that reflects the underlying degradation state of the system.

Despite the strong representational capacity of the Transformer, the initial supervised predictions may still exhibit residual errors due to non-stationary operating conditions, sensor noise, or model bias. To mitigate these limitations, the proposed methodology integrates an RL-based refinement stage. In this formulation, the prediction error correction is cast as a policy optimization problem, where an RL agent learns an adaptive adjustment factor aimed at minimizing a predefined cost function associated with RUL estimation errors.

### 3.1. Proposed Hybrid Architecture

The utilization of a two-stage hybrid architecture, meticulously designed to enhance the accuracy of RUL prediction in turbofan engines, is hereby proposed. This approach will be implemented through the employment of the Commercial Modular Aero-Propulsion System Simulation (CMAPSS) dataset [58], a comprehensive collection of data that has been instrumental in the field. This configuration integrates the time series modeling capability of the Transformer with the fine-tuning accuracy of a reinforcement learning agent, specifically the TD3 algorithm. The Transformer is employed as the baseline predictor, responsible for extracting high-level representations. In contrast, TD3 operates as an error mechanism, guided by a particular reward law, with the objective of adjusting predictions to minimize the root mean square error (RMSE).

**Figure 4:**
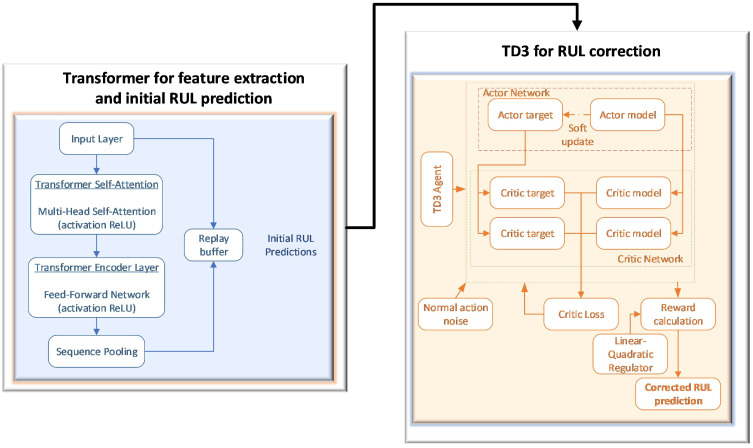
Proposed hybrid architecture.

Figure 4 illustrates the architectural framework employed in this study.

#### 3.1.1. Feature Extraction and Baseline Reduction with Transformer

The base model is a Transformer encoder architecture adapted for regression tasks. The objective of this stage is to learn the temporal dependencies and relationships between sensors within data sequences to generate an initial estimate of the RUL for each motor at the end of its test history.

The data from the CMAPPS dataset is normalized, and fixed-length sequences are defined to feed the model:

(16) $$X_{T}=Y_{T+seqlen}$$

In this particular case, $X_{T}$ denotes the input sequence corresponding to target RUL value $Y_{T+seqlen}$.

The Transformer comprises an input layer (*nn.linear*), which functions to project the input features into a latent space of $d_{model}$ dimensions. This step is of paramount importance for model integration, as it prepares the sequence for self-attention processing.

Each layer of the Transformer encoder ($num_{layers}$) incorporates a multi-head self-attention mechanism with *nhead* heads and a *Feed-Forward* network with a considerably larger dimension.

The output of the final layer of the transformer encoder is derived exclusively from the final position of the sequence (X[:,−1,:]), thereby compressing the temporal information of the sequence into a single vector. This approach is predicated on the premise that the most recent encoded representation is the most pertinent to the present state of engine degradation.

The output network (*head*) is processed by an output network (*nn.sequential*) consisting of a hidden layer followed by a final linear layer to a neuron (scalar prediction of RUL). Transformer training is performed using the mean squared error criterion (*MSELoss*) as the loss function.

#### 3.1.2. Correction of Predictions Through Reinforcement Learning (TD3)

A customized environment is thus established, predicated on the Transformer’s predictions and the actual RUL values. The agent systematically processes each prediction for each motor.

The observation space is defined as a fixed-dimension vector containing the transformer’s prediction for the current motor $P_{t}$ and the RUL of that motor $RUL_{real}$. The incorporation of the RUL into the state enables the agent to assess its present performance and make informed correction decisions during training.

The action space is defined as a continuous scalar value (dimension 1) that represents the correction proposed by the agent. The action range is constrained by a lower limit and an upper limit, thereby modulating the intensity of the corrective actions. The reward and penalty law is defined to maximize the proximity of the corrected prediction to the actual value.

TD3 is configured with an MLP (Multi-Layer Perceptron) policy for its actor and critic networks. To promote the exploration of corrections during training, normal action noise with an optimized standard deviation σ is employed.

Training hyperparameters include the implementation of several key elements, including the configuration of a low learning rate (*lr*), the establishment of an ample repeat buffer size, a typical batch size, a small smoothing factor τ for target networks, and a discount factor γ close to 1.

#### 3.1.3. Interconnection and Final Prediction

The proposed hybrid approach is predicated on a cascade architecture. Initially, the Transformer is trained to generate the baseline RUL prediction for each test motor. These predictions are then saved to feed the TD3 environment. Subsequent to the training of the TD3 to generate the ideal corrective action, the ultimate RUL is ascertained.

This architectural design enables the customized optimization of each component. The Transformer is capable of focusing on learning robust representations of the motor state, while the TD3 specializes in minimizing residual error. This allows the final model to combine the strengths of deep sequential modeling and reward-based optimization.

### 3.2. Transformer Parameters

Hyper-optimization of the Transformer model is a crucial process that directly impacts its ability to learn complex temporal dependencies and degradation characteristics in the CMAPSS dataset. It is imperative to adjust the hyperparameter of this architecture, particularly those of the encoder block, to circumvent the pitfalls of overfitting or underfitting, especially in light of the sequential and noisy nature of sensor data.

Table 2 presents the parameters that were selected for optimization, along with the values employed in the hyper-optimization process.

A pivotal parameter in this regard is the model dimension (*dim model*), which stipulates the extent of the latent space into which the input features are projected. The dimension should be sufficiently large to capture intricate patterns without being excessively large, as this would unnecessarily increase computational cost. The number of attention heads (*num head*) is a critical factor in determining the extent to which the self-attention mechanism explores independent representation subspaces. It is standard practice to set the *dim model* so that it is divisible by nhead to ensure efficient division in multi-head attention calculations.

The number of encoder layers (*num layers*) is a critical factor in determining the model’s dimension and its capacity to learn feature hierarchies. A more complex model, comprising additional layers, enables the refinement of representations through multiple iterations of self-attention and feed-forward propagation, thereby enhancing the extraction of complex degrading features. However, an increase in depth concomitantly increases the risk of training instability and convergence time. Therefore, the selection of this method must strike a balance between representational capacity and training stability.

Within each layer of the Transformer, the dimension of the feed-forward network (*dim feedforward*) is of paramount importance. This internal dimension is generally larger than the *dim model* and enables the application of complex nonlinear transformations to the representations generated by attention. It is imperative to adjust this parameter, as an insufficient mapping capacity can be indicative of an overly diminutive value, whereas an excess of redundancy can impede the efficiency of training.

The length of the input sequence (*sequence length*) is a critical hyperparameter specific to time series. A longer *sequence length* enables the model to observe longer-term degradation trends, which is pertinent for RUL. However, this also results in a substantial increase in memory costs and the quadratic complexity of the self-attention calculation. In the context of machine learning algorithms, the *learning rate* has been demonstrated to play a pivotal role in the process of convergence. In many cases, it is imperative to employ a low *learning rate* in conjunction with an adaptive optimizer, such as the Adam optimizer, to ensure the stability and efficacy of the training process. The *batch size* exerts a direct influence on the accuracy of the gradient estimate and memory consumption. A moderate value is selected to achieve a balanced ratio between training speed and learning quality.

The number of encoder layers (*num layers*) was constrained to shallow and moderately deep configurations to circumvent excessive instability during training while enabling hierarchical feature learning. The dimension of the feedforward network (*dim feedforward*) was explored with values commonly used in industrial time series applications to introduce sufficient nonlinearity without significantly increasing computational cost. The *sequence length* was selected to encompass short- and medium-term degradation dynamics, acknowledging the quadratic complexity of self-attention with respect to sequence length. The *learning rate* range was finally defined to ensure stable convergence when using adaptive optimizers, such as Adam. This range was limited to values reported as effective in similar studies of transformer-based forecasts. These criteria ensure that the hyperoptimization process is reproducible and aligned with established best practices.

The parameters displayed in Table 3 represent a judicious compromise between the sequence model’s capacity and computational efficiency, with a compact yet efficient architecture for the Transformer. The Optuna library was utilized for automated and iterative hyperparameter search for each of the subsets of the CMAPSS dataset.

In the context of replication tasks, it is imperative to acknowledge that the set of hyperparameters selected for optimization in Table 3 was defined based on previous research on RUL prediction using Transformer architectures and practical considerations related to the model’s capacity, stability, and computational efficiency. The embedding dimension (*dim model*) was modified to achieve a balance between representation power and the risk of overfitting. Specifically, low values were found to favor compact models, while high values were conducive to more extensive feature extraction. The number of attention heads (*num head*) was selected so that the *dim model* is divisible by *num head*. This ensures efficient multi-head attention computation and allows the model to capture diverse temporal relationships across sensor channels.

### 3.3. Environment Used in TD3

The implementation of TD3 necessitated the development of a customized reinforcement learning environment, designated as RULCorrectionEnv, to facilitate the learning process. This environment was meticulously engineered to enhance the efficacy of the RUL predictions previously derived from the Transformer model. In contrast to conventional RL environments, where the agent engages with a physical or simulated system, this environment encapsulates the error process by leveraging the outputs of a pre-trained machine learning model and using the outputs of a pre-trained machine learning model and the values of the test set as a basis.

The design of the environment is predicated on the transformation of the regression refinement problem into a sequential decision-making problem. The TD3 agent does not receive the raw characteristics from the engine; rather, it operates on a simplified state composed of the prediction and the target value (actual RUL). This formulation enables the agent to prioritize the development of a π(s) adjustment policy that aims to minimize the discrepancy between the corrected prediction and the actual value. This approach obviates the necessity of relearning the intricate sequential dynamics of the sensor data.

The interaction between the agent and the environment is governed by the precise definition of the state space and the reward law. The agent’s action constitutes a continuous value that signifies the extent of the adjustment to the Transformer’s prediction. Conversely, the reward is identified as the pivotal metric that directs learning.

The customized environment for TD3 is structured as follows:

#### 3.3.1. State

Take into account the current Transformer prediction and progress in the dataset (normalized index), as can be seen in Equation (17).

(17) $$s_{t}=\left[{\hat{y}}_{t},\frac{t}{N}\right]$$

where ${\hat{y}}_{t}$ is the transformer’s prediction, and *N* is the total number of samples at time *t*.

#### 3.3.2. Action

The agent decides on a correction $a_{t}$ within the defined range. The corrected prediction can be seen in Equation (18).

(18) $${\hat{y}}^{corr}=\hat{y}+a_{t}$$

#### 3.3.3. Reward Law

It measures the difference between the corrected prediction and the actual value of the RUL. The recompense is negative of the absolute error as seen in Equation (19).

(19) $$r_{t}=-\left|{\hat{y}}^{corr}-y_{t}\right|$$

The reward can be interpreted as a typical cost criterion, as observed in Equation (20).

(20) $$r_{t}=-\left|e_{t}\right|$$

This is equivalent to minimizing the cost function, as can be seen in Equation (21).

(21) $$J=\sum_{t=0}^{T}\left|e_{t}\right|$$

Considering a traditional linear quadratic regulator (LQR):

(22) $$J_{LQR}=\sum_{t=0}^{T}\left(x_{t}^{T}Qx_{t}+u_{t}^{T}Ru_{t}\right)$$

LQR has a quadratic cost function. The reward is defined in Equation (23).

(23) $$r_{t}=-\left(e_{t}^{2}+\lambda a_{t}^{2}\right)$$

where:

- $e_{t}^{2}$ penalizes the quadratic error of accuracy.
- $\lambda a_{t}^{2}$ penalizes the control effort (to avoid excessive or unstable corrections).
- λ>0 is the weighting factor between accuracy and effort.

This corresponds to the minimization of the cumulative cost, which has an LQR structure, as can be seen in Equation (24).

(24) $$J=\sum_{t=0}^{T}\left(e_{t}^{2}+\lambda a_{t}^{2}\right)$$

- The state $x_{t}^{T}$ is represented as the error $e_{t}^{2}$.
- The control input $u_{t}^{T}$ is equivalent to the action $a_{t}^{2}$.
- Q=1 and R=λ are used, which is a scalar version of LQR.

The selection of a reward function based on the LQR architecture is justified by the need to transform the RUL refinement process into a stable control problem, based on the following technical considerations:

- Achieving an equilibrium between precision and stability is imperative. The LQR structure enables the model to minimize the quadratic error of accuracy ($e_{t}^{2}$) while concomitantly penalizing the “control effort” or magnitude of the correction ($\lambda a_{t}^{2}$). This prevents unstable or erratic adjustments.
- Smooth Policy Convergence Contrary to conventional reward functions that prioritize the final error, the quadratic nature of LQR guarantees a more refined learning policy. This is due to the fact that large deviations are heavily penalized, and corrections become more precise as the agent approaches the target RUL.
- Robustness versus noise. In complex scenarios, such as those observed in the FD002 and FD004 datasets, the LQR-based reward functions as a dynamic regulator, attenuating oscillations caused by sensor noise or non-stationary operating conditions.
- Mathematical Consistency. This formulation facilitates the minimization of a cumulative cost function (*J*), thereby providing a mathematically well-founded framework that aligns the reinforcement learning objective with classical control theory for predictive maintenance.

As with the Transformer, some parameters of the TD3 underwent a hyper-optimization process, which can be seen in Table 4.

The efficacy of the TD3 agent in the task of RUL correction is contingent upon the optimization of its parameters, which govern the reinforcement learning process and the dynamics of error correction. Parameters such as *learning rate*, *buffer size*, and *batch size* are foundational to the stability and convergence speed of the actor and critic networks. A low learning rate is essential for ensuring smooth and controlled weight updates, thereby preventing divergences. Additionally, a large buffer size is crucial for the storage of diverse correction experiences, which is vital for TD3 off-policy learning. The batch size, which is defined as the sample size used for each gradient update, has been shown to affect the noise in the gradient estimation.

It is imperative to consider other pivotal parameters that delineate the trade-off between exploration and exploitation, in addition to the stability of learning. The discount factor (*gamma* γ), which is generally set close to unity, determines the importance of future rewards and is essential for assessing the long-term consequences of corrections. The smoothing factor, designated as *tau* (τ), governs the updating of target networks. A low value of tau is imperative for preserving the stability of the algorithm, as it ensures gradual updates. Conversely, the Gaussian noise value (*noise sigma*) impacts the exploration of the continuous action space, enabling the agent to attempt diverse corrections and circumvent the formation of local optima during the training process.

The parameters *lambda penalty*, *action low*, and *action high* are unique to the created environment and delineate the correction policy. The action range, defined as the extent of possible actions within a given context, serves to constrain the magnitude of corrections that the agent is capable of implementing, thereby directing its concentration. The *lambda penalty* (λ) is a term used to describe the impact of the size of the action in the reward function. It has been demonstrated to encourage minimal and stable adjustments. Ensuring the generalization and robustness of the model necessitates the consistent application of the same parameter values to the four CMAPSS subsets (FD001, FD002, FD003, FD004). This approach aims to demonstrate that the TD3 model, once optimized, maintains its correction effectiveness across a range of operating and fault conditions.

### 3.4. Data and Preprocessing

The dataset CMAPSS, produced with NASA’s turbofan engine simulation tool, contains a series of multivariate time-series measurements [58]. In each trajectory, the engine begins operating under normal conditions, but at a specific moment, a fault is introduced. This defect gradually intensifies throughout the sequence until the engine reaches complete failure.

**Figure 5:**
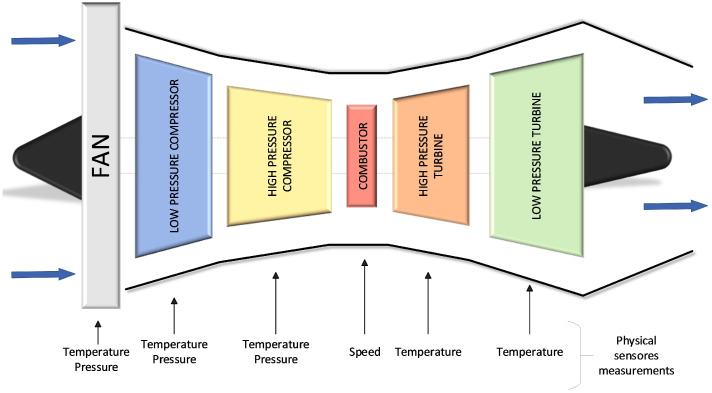
Turbofan structure.

As shown in Figure 5, the turbofan engine includes several major subsystems, such as the high- and low-pressure compressors, the high- and low-pressure turbines, and the combustion chamber.

The turbofan engine is equipped with 58 sensors for routine performance monitoring; only 21 of these measurements are retained in the dataset, as they are deemed dependable. The CMAPSS dataset is divided into four subsets, each containing separate training and testing partitions. These subsets vary in their operating conditions and failure configurations, leading to a gradual increase in complexity from FD001 through FD004. A detailed summary of these subsets is provided in Table 5.

The training sets include RUL sequences in which engines are monitored from healthy operation through progressive degradation. Each entry comprises 26 attributes: the engine ID, the time cycle, six operational parameters, and 21 sensor readings. In addition, the dataset incorporates six distinct operating conditions and two specific failure mechanisms—fan deterioration and high-pressure compressor degradation.

Table 6 provides a comprehensive overview of the nomenclature, technical description, and units of measurement for the 21 sensor variables monitored in the turbofan engine, in accordance with the standard configuration of the C-MAPSS dataset. These measurements, which encompass critical parameters of temperature, pressure, and rotational speed in various engine components, constitute the input space for the TRANS-D3 model in the regression task for RUL prediction.

All CMAPSS subsets were utilized in the experiments presented in this study. Prior analyses have shown that several features in the dataset contribute minimally to RUL prediction. Following the recommendations in [59], a set of 14 features was selected for use in the proposed hybrid model. These correspond to columns 2, 3, 4, 7, 8, 9, 11, 12, 13, 14, 15, 17, 20, and 21. Moreover, the sensor data are scaled using min–max normalization, which is defined by the following expression [42]:

(25) $$x^{\prime }=\frac{x-x_{min}}{x_{max}-x_{min}}$$

In Equation (25), *x* represents the raw sensor measurement, while $x_{min}$ and $x_{max}$ correspond to the minimum and maximum observed values of that sensor, respectively. Applying this normalization maps all sensor readings to the interval [0, 1], ensuring consistency across features and aiding the training process in producing reliable RUL estimation models. Additionally, using a refined set of sensors together with normalization has been shown to improve both the accuracy and stability of the predictive models [60].

**Figure 6:**
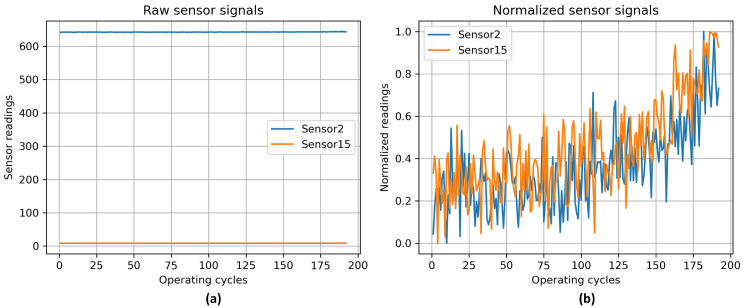
Comparison of (**a**) raw and (**b**) normalized sensor signals (Sensor 2 and Sensor 15).

To facilitate an intuitive comprehension of the behavior of each sensor and the effect of min–max normalization, Figure 6 illustrates representative raw and normalized signals from selected sensors.

To illustrate this point, two sensors were selected for analysis: Sensor 2 and Sensor 15. These sensors were chosen to demonstrate the impact of the min–max normalization process on the raw degradation signals. The sensors exhibit varying dynamic ranges and degradation trends, rendering them well-suited for elucidating the process of normalization, which rescales heterogeneous sensor measurements to a shared numerical range while preserving their temporal progression. As demonstrated in Figure 6, the implementation of preprocessing techniques effectively mitigates scale disparities and enhances numerical stability without compromising the integrity of underlying degradation patterns. The same normalization strategy is systematically applied to all sensor channels and consistently adopted across all CMAPSS subsets (FD001–FD004), ensuring homogeneous preprocessing and fair model training and evaluation conditions.

### 3.5. Hardware Used

The development was performed on a laptop featuring an 11th-generation Intel Core i7 CPU, an NVIDIA RTX 3060 GPU, and 16 GB of RAM. The hybrid model was implemented using the Python 3.11 programming language.

## 4. Results

This section provides a thorough evaluation of the Trans-D3 hybrid architecture’s performance in predicting RUL using the CMAPPS dataset. The results are organized to show the effectiveness of the proposed approach compared to other algorithms used in RUL prediction.

### 4.1. Metrics

Two important metrics are used to evaluate the performance of the proposed hybrid approach in predicting RUL: RMSE and score. As shown in Equation (26) [61], RMSE is a commonly used metric in RUL evolution that assigns the same weight to underestimation and overestimation of RUL. Here, $y_{i}$ are the actual values and $y_{i}^{\prime }$ are the predicted values in the $i_{th}$ cycle. The lower RMSE value represents better accuracy.

(26) $$RMSE=\sqrt{\frac{1}{n}\sum_{i=1}^{n}{\left(y_{i}-y_{i}^{\prime }\right)}^{2}}$$

On the other hand, the Score defined in Equation (27) introduces different types of penalties depending on whether the prediction is late or early [58].

(27) $$SCORE=\left\{\begin{array}{cc} \sum_{i=1}^{n}e^{-}\frac{y_{i}-y_{i}^{\prime }}{13}-1, & d<0\\ \sum_{i=1}^{n}e^{\frac{y_{i}-y_{i}^{\prime }}{10}}-1, & d\geq 0\; \end{array}\right.$$

**Figure 7:**
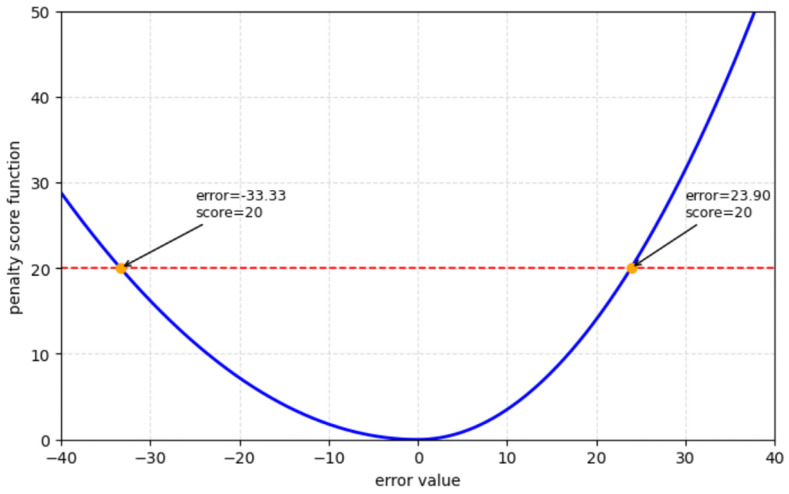
Penalty score function.

Here, $y_{i}-y_{i}^{\prime }$ denotes the difference between the true RUL $y_{i}$ and the predicted value $y_{i}^{\prime }$ at the *i*-th cycle. The score function assigns penalties to both overly optimistic and overly conservative estimates. When the model outputs a smaller RUL than the actual value (early prediction), the resulting penalty is relatively small. Conversely, when the predicted RUL is greater than the true value (late prediction), the penalty becomes more substantial, as failing to anticipate an imminent failure can pose serious risks for equipment reliability and maintenance scheduling. As illustrated in Figure 7, the tolerance for advanced prognostics is observed to be greater than that for delayed prognostics, given a constant penalty score value.

### 4.2. RUL Prediction

The performance obtained by the hybrid approach proposed in this paper is compared with other algorithms used for the same task found in the SOTA. These methods include support vector regression (SVR) [62], MLP [62], CNN [62], LSTM [62], CNN + LSTM [62], bidirectional LSTM [63], gated convolutional transformer (GCT) [32], deep convolutional neural networks (DCNN) [59], ensemble LSTM neural network (ELSTMNN) [64], distributed attention-based convolutional network (DATCN) [65], gated recurrent unit convolution neural network (AGCNN) [66], BiLSTM attention model [17], dual aspect self-attention (DAST) [67], dynamic length Transformer (DLformer) [33], 1D-CNN-LSTM [68], CNN-LSTM-Self-Attention Mechanism (SAM) [34], BiLSTM-Denoising Autoencoder (DAE) [35]-Transformer, and Two-Stage Attention-Based Hierarchical Transformer (STAR) [39].

All comparative results were obtained from original publications or benchmark evaluations conducted according to the standard CMAPSS protocol. These evaluations employed identical training/test splits and metrics, ensuring a fair and consistent comparison.

To ensure replicability, please refer to Table 3 and Table 4, which list all parameters used for TRANS-D3. Additionally, the same min–max normalization was applied to scale the sensor readings to the interval [0,1], using exclusively the standardized subset of 14 sensors (columns 2, 3, 4, 7, 8, 9, 11, 12, 13, 14, 15, 17, 20, and 21) recommended in the literature for the CMAPSS dataset, which were used in the works used for comparison.

As can be seen in Table 7, the bold number represents the best model.

The proposed TRANS-D3 model demonstrates a substantial enhancement in the FD001 dataset, achieving an RMSE of 1.66, which signifies an 84–90% reduction in error compared to advanced models such as STAR (10.61), BiLSTM-DAE-Transformer (10.98), or AGCNN (12.42). In terms of the score, the improvement is even more marked. While traditional methods register values between 169 and 3980, the proposed method obtained 8.82, which is equivalent to a reduction of more than 95%. This suggests that not only is there lower error, but also a very low penalty for more accurate and conservative predictions.

In the case of the FD002 dataset, the proposed TRANS-D3 model also demonstrates significant enhancements in comparison to the majority of the comparative models. With an RMSE of 13.23, the model outperforms architectures such as DATCN (16.95), AGCNN (19.43), DAST (15.25), and CNN-LSTM-SAM (18.9), achieving an error reduction of between 14% and 32%. Despite the noteworthy performance of certain specialized models, such as STAR (13.47) or BiLSTM Attention (15.94), TRANS-D3 remains a notable outlier with an impressive score of 114.33. This result indicates a substantial reduction of 85–95% compared to methods like BiLSTM-DAE-Transformer (2937), AGCNN (1492), DATCN (1842), or STAR (784). This finding indicates that, even in the most intricate scenarios characterized by operational variability across units, the TRANS-D3 hybrid approach consistently generates more stable predictions, accompanied by reduced penalty and enhanced generalization capability.

In FD003, TRANS-D3 is also identified as one of the most competitive models, with a reduction in RMSE to 6.61, representing an improvement of 38–45% over advanced networks such as BiLSTM-DAE-Transformer (11.14) or DATCN (11.56), and more than 50% compared to traditional architectures such as CNN (21.36) or MLP (31.52). In terms of the Score metric, the model achieves a value of 42.88, indicating an approximate reduction of 80–85% compared to methods such as CNN-LSTM-SAM (253) or AGCNN (227). This finding serves to substantiate the efficacy of the TRANS-D3 hybrid in discerning subtle degradation patterns in engines confronted with intricate operating conditions.

In FD004, which has been identified as challenging due to its high variability, the proposed model achieves an RMSE of 12.26, representing improvements between 23% and 35% over recent models such as STAR (15.87), CNN-LSTM-SAM (20.5), and DATCN (18.23). The Score metric demonstrates a comparable effect: while the comparative methods range from 1449 to 3392, TRANS-D3 registers 406.93, implying a 70–85% reduction in penalty. The findings of this study demonstrate that the hybrid approach integrating Transformer and TD3 not only enhances accuracy but also substantially augments prediction stability in scenarios characterized by high uncertainty.

In order to provide a more comprehensive statistical validation of the TRANS-D3 model and to characterize the degree of linear association and fit between estimated and actual RUL values, Table 8 summarizes the coefficient of determination ($R^{2}$), Pearson’s correlation coefficient (*r*), and 95% confidence intervals for the prediction error for each CMAPSS subset.

In addition to the RMSE and score metrics presented in Table 7, a more in-depth statistical evaluation was performed to assess the model’s reliability in Table 8. The TRANS-D3 model achieved an $R^{2}$ greater than 0.93 in all subsets, with a maximum value of 0.9984 for FD001. This indicates that the hybrid architecture explains nearly all the variance in RUL degradation trajectories. Furthermore, the coefficient *r* remained above 0.96 in all scenarios, confirming high linear consistency between correct predictions and actual results, even under the multi-regime operating conditions of FD002 and FD004.

**Figure 8:**
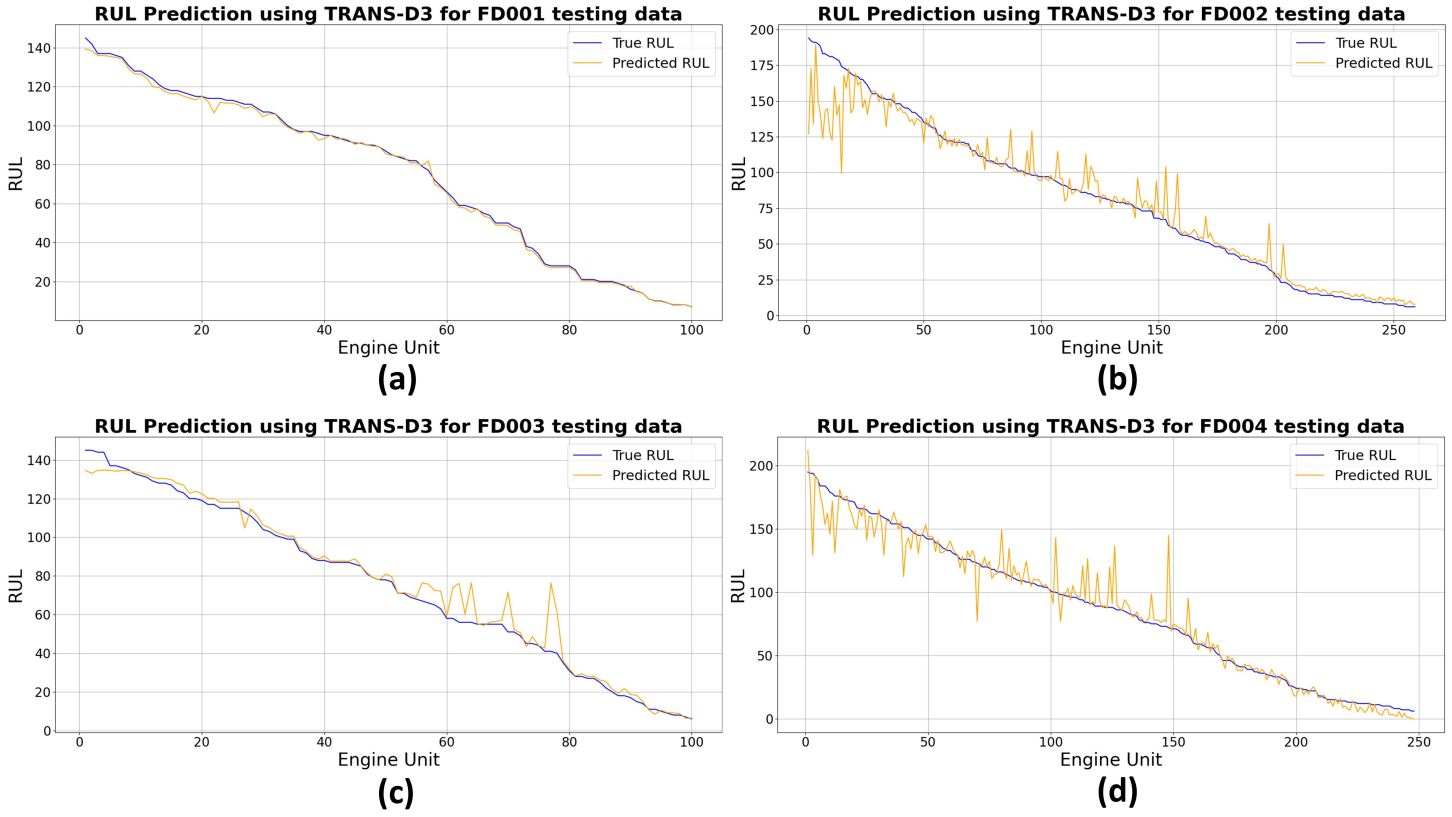
Results comparing the estimated RUL with the actual RUL. Comparison results for (**a**) FD001; (**b**) FD002; (**c**) FD003; (**d**) FD004.

Figure 8 presents a detailed visual comparison between the proposed TRANS-D3 model and the actual RUL from the test dataset FD001 to FD004. The *x*-axis is thus directly proportional to the motor unit index, whilst the *y*-axis is inversely proportional to the RUL.

Figure 8 illustrates the comparison between the True RUL, plotted in blue, and the Predicted RUL. The motor units on the X-axis have been ordered from highest to lowest True RUL in order to visualize the trend. The objective of the correction is to align the prediction with reality, and the outcome is evident: the orange line (Corrected RUL) in all datasets closely mirrors the trend and values of the blue line (Actual RUL). The proximity of both curves throughout the entire range, from the units with the highest RUL (left) to those with the lowest RUL (right), indicates that the correction strategy employed by TRANS-D3 has been highly effective in significantly reducing the prediction error in the analyzed sample, although some slight deviations are observed that suggest a small residual error.

**Figure 9:**
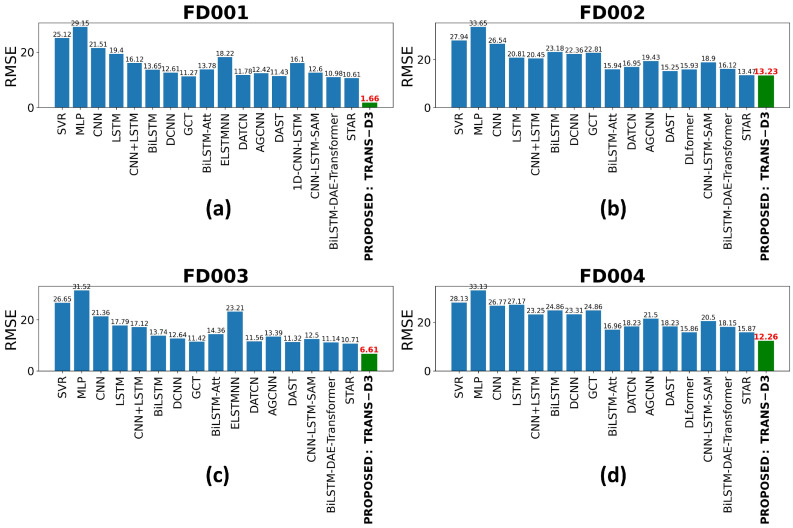
RMSE results for (**a**) FD001; (**b**) FD002; (**c**) FD003; (**d**) FD004.

Figure 9 presents a comprehensive comparison of the RMSE between various SOTA models and the proposed method, TRANS-D3, evaluated on the four subsets of the C-MAPSS benchmark (FD001 to FD004). The findings indicate that the proposed architecture consistently surpasses all reference models, including those based on recurrent neural networks (LSTM, BiLSTM), convolutional networks (CNN, DCNN), and hybrid attention models (BiLSTM-Att, CNN-LSTM-SAM). A substantial decrease in error is evident across all scenarios, particularly in FD001, where TRANS-D3 attains an RMSE of 1.66, signifying a notable enhancement in prediction accuracy when compared to the STAR model (10.51) and alternative contemporary methods.

This superior performance is maintained even in the FD002 and FD004 subsets, which are more complex due to multiple operating conditions and failure modes. Conventional models, such as the MLP and the SVR, demonstrate a substantial decline in accuracy, with RMSE values surpassing 25 or 30 points. Conversely, the TRANS-D3 model exhibits exceptional resilience, maintaining the lowest errors in the dataset (13.23 for FD002 and 12.26 for FD004). These findings suggest that the integration of attention mechanisms and the structure of the proposed model allows for more effective temporal feature extraction, achieving exceptional generalization for RUL prediction in complex industrial systems.

**Figure 10:**
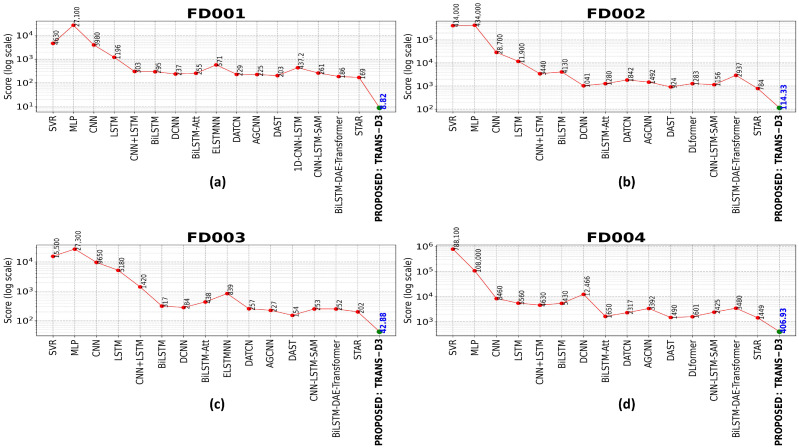
Score results (log scale) for (**a**) FD001; (**b**) FD002; (**c**) FD003; (**d**) FD004.

As illustrated in Figure 10, a comparison of performance in terms of score functions is presented for the four subsets of the C-MAPSS dataset. Due to the nature of this metric, which asymmetrically penalizes overestimation errors in the RUL forecast, the resulting values show significantly different orders of magnitude between conventional models and the proposed model. Consequently, a logarithmic scale has been employed on the y-axis. This representation is essential for facilitating a clear and equitable visualization of performance differences, thereby preventing outliers from low-performing models (e.g., MLP or SVR) from obscuring the subtle yet critical improvements achieved by more advanced architectures.

The implementation of this visualization technique has enabled the discernment of TRANS-D3’s noteworthy advancements, evidenced by its positioning at the lower end of the error scale across all evaluated scenarios. Within the FD001 subset, the proposed model attains a value of 8.82, a result that positions it considerably below the majority of documented models in the scientific literature, which frequently surpass the $10^{2}$ and even $10^{3}$ threshold. This tendency towards superiority is corroborated in more complex environments, such as FD002 and FD004, where TRANS-D3 attains scores of 114.33 and 106.93, respectively. The model’s capacity to sustain such minimal errors on a logarithmic scale accentuates its robustness and efficacy in capturing intricate temporal dependencies, thereby minimizing critical safety failures associated with overestimating the service life of mechanical components.

**Figure 11:**
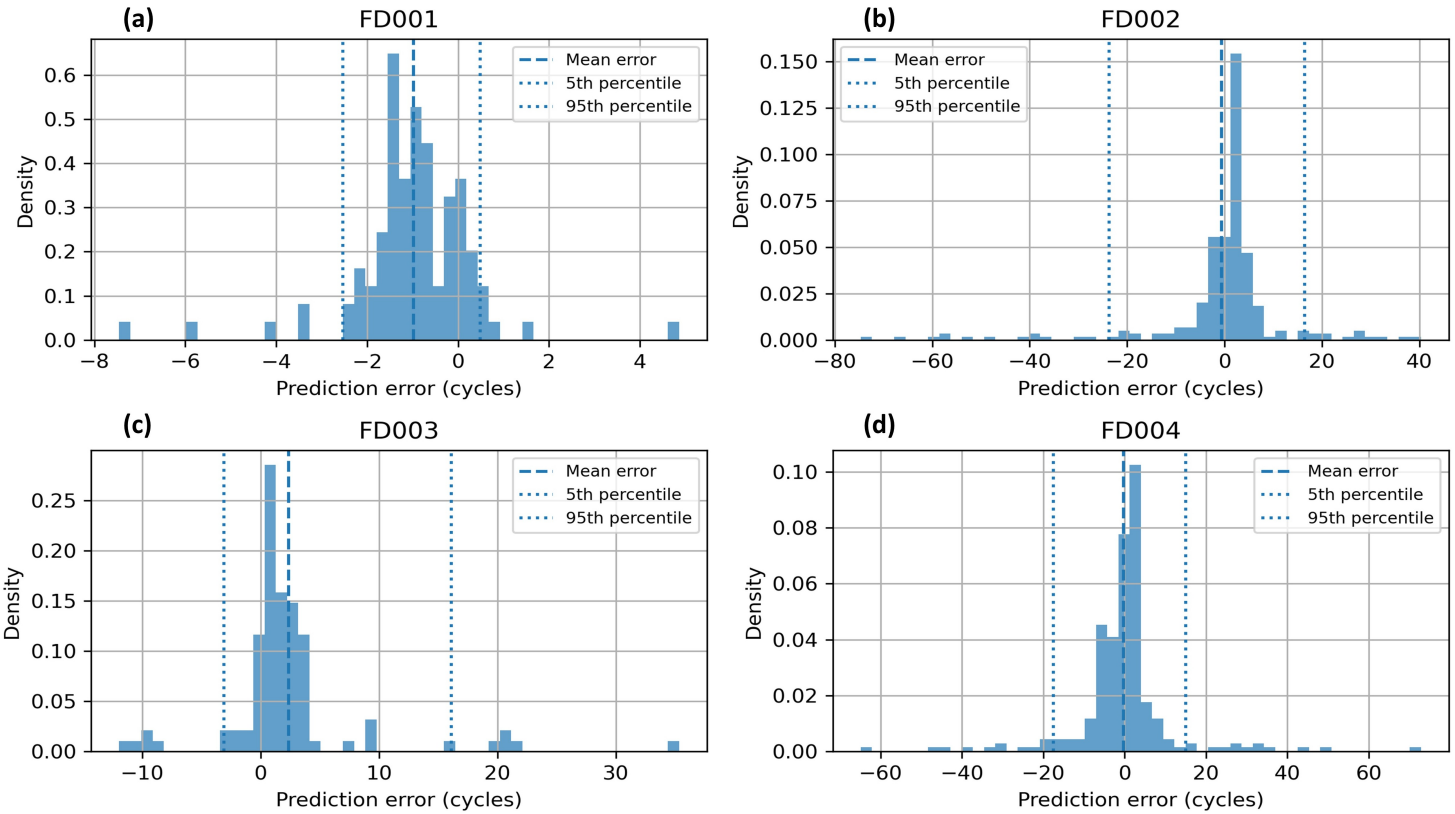
Distribution of the prediction error density of the RUL for (**a**) FD001; (**b**) FD002; (**c**) FD003; (**d**) FD004.

Furthermore, the robustness and dispersion of the predictions must be thoroughly evaluated. As illustrated in Figure 11, the density distribution of the prediction error (in cycles) for the four CMAPSS subsets is shown, with percentile-based confidence intervals used to quantify the uncertainty of the model.

Figure 11 presents the uncertainty analysis using the prediction error distribution, comparing the model estimate with the actual RUL values. An analysis of the four scenarios (FD001–FD004) reveals a notable concentration of errors around the zero value, as indicated by the mean error line. The unimodal and centered distribution indicates that the model exhibits minimal systematic biases, ensuring consistent accuracy across complex subsets such as FD002 and FD004, which are characterized by multiple operating conditions and failure modes.

To provide a quantitative measure of reliability, the 5th and 95th percentiles (dotted lines) have been calculated, delineating the range in which 90% of the predictions are found. This approach to quantifying uncertainty enables the observation of the sensitivity of the model to different environments. While in FD001 the dispersion is minimal (indicating high certainty), in FD002 and FD004 the intervals widen. This behavior is consistent with the CMAPSS literature and demonstrates the model’s capacity to recognize and represent the inherent variability of sensor data under fluctuating operating conditions, thereby providing an explicit confidence margin for the diagnosis.

From a predictive maintenance perspective, these results validate the reliability of the proposed system. The establishment of statistical limits for prediction error enables maintenance operators to ascertain not only the estimated RUL but also the associated risk level. The low density of outliers at the extremes of the graphs confirms that the model maintains its stability, reducing the probability of critical prediction errors.

In order to provide a more comprehensive overview of the uncertainty analysis, Table 9 presents a summary of the primary statistical indicators of the prediction error distribution in the CMAPSS datasets.

The results presented in Table 9 offer a quantitative characterization of the prediction error distribution across the four CMAPSS subsets. The mean error values are found to be close to zero in all cases, indicating that the proposed TRANS-D3 framework does not demonstrate a systematic bias toward overestimating or underestimating the RUL. Specifically, the FD001 subset demonstrates minimal error variation, indicative of the stable operating conditions within this scenario. This finding validates the efficacy of the proposed method in generating reliable and precise predictions under nominal conditions.

Conversely, the higher standard deviation and wider percentile ranges observed in FD002, FD003, and FD004 are consistent with the greater operational variability and fault complexity inherent in these subsets. Notwithstanding the aforementioned challenges, TRANS-D3 exhibits controlled error dispersion and limited extreme deviations, as evidenced by the 5th and 95th percentiles. This behavior underscores the robustness of the proposed control-oriented learning strategy and its aptitude for real-world predictive maintenance applications, wherein the reliability and stability of predictions are paramount for decision-making in Industry 4.0 environments.

It is imperative to draw a distinction between uncertainty analysis and the statistical confidence intervals that are provided. The 5th and 95th percentiles delineate the dispersion of individual prediction errors in the test engines shown in Table 9. Conversely, the 95% confidence intervals (CI) in Table 8 refer to the precision of the mean error estimate. The observation that the confidence interval of the mean error remains close to zero in all subsets provides evidence that the TRANS-D3 framework is an unbiased estimator. This dual approach, which involves the quantification of both the dispersion of individual predictions and systematic bias, provides a comprehensive profile of model reliability in Industry 4.0 environments.

To further investigate the adaptability and resilience of the proposed TRANS-D3 framework, an additional evaluation was performed using a different dataset than the one used during training, specifically a variant of CMAPSS (N-CMAPSS) [69]. While classic CMAPSS datasets remain the primary reference and de facto standard in the literature for validating new RUL prediction methodologies, evaluating performance with an independent and more recent dataset is essential to verify that the proposed approach is not limited to a single data source. The objective of this complementary analysis is to demonstrate the ability of TRANS-D3 to generalize its predictive and corrective mechanisms under heterogeneous operating conditions and unobserved data distributions.

The N-CMAPSS dataset has been meticulously engineered to emulate a high-fidelity simulation of turbofan engine degradation under realistic operating conditions. It encompasses multiple flight profiles, time-varying operating regimes, and complex degradation trajectories that evolve over the engine’s lifecycle. The dataset under consideration captures non-stationary sensor responses, variable noise levels, and complex interactions between operating settings and sensor measurements. These characteristics introduce significant temporal and contextual complexity, making N-CMAPSS an ideal benchmark for evaluating the robustness and adaptability of data-driven predictive models in realistic industrial environments.

The experimental results obtained in the N-CMAPSS DS01 subset confirm that TRANS-D3 retains its robustness and stability under these more demanding conditions. The observed performance demonstrates that the proposed framework is not dependent on specific adjustments for each dataset, as long as the model architecture and hyperparameters used are consistent with those employed in classic CMAPSS experiments. Conversely, the integration of Transformer-based temporal representation with TD3-based error correction enables the model to effectively adapt to greater operational variability and complex degradation patterns.

To provide a comprehensive quantitative evaluation of TRANS-D3 on the N-CMAPPS DS01 dataset, the same metrics used previously are employed, as can be seen in Table 10.

Table 10 offers a synopsis of the TRANS-D3 framework’s predictive capabilities when applied to the DS01 subset of N-CMAPSS. The model demonstrated a low RMSE of 3.57 cycles, indicating an accurate estimation of RUL at the unit level. In addition, the SCORE value of 64.13 indicates a minimal penalty for both early and late predictions, thereby underscoring the robustness of the proposed correction strategy when evaluated under a risk-sensitive framework. Conversely, the high coefficient of determination ($R^{2}=0.9624$) substantiates that TRANS-D3 effectively captures the degradation dynamics of the system and elucidates a substantial proportion of the variance in RUL trajectories. The findings demonstrate the efficacy of the proposed hybrid reinforcement learning and Transformer architecture in delivering reliable and stable forecasting performance on the N-CMAPSS dataset.

## 5. Discussion

The integration of the Transformer model with the TD3 algorithm resulted in substantial enhancements in RUL prediction in comparison with conventional supervised methodologies. The cascade structure enabled each component to solve a specific aspect of the problem. The Transformer focused on learning degradation patterns from multivariate time series, while TD3 systematically reduced residual error through a reward-based optimization process. This synergy is a primary factor in the observed increase in final accuracy.

One of the most relevant aspects of the results is that the Transformer effectively captures the nonlinear variations in the sensors on its own but shows increasing bias in the later stages of degradation. This behavior is common in purely supervised models because the data distributions in the final phases are more dispersed and underrepresented. Incorporating TD3 made it possible to correct these deviations by formulating them as a control problem in which the agent regulates an adjustment that reduces the RMSE.

The comparative results presented in Table 7 demonstrate that Trans-D3 consistently outperforms or matches recent SOTA approaches on the CMAPSS benchmark, both in terms of RMSE and the score. Specifically, the proposed framework demonstrates significant advancements over purely supervised deep learning models, such as CNN- and LSTM-based architectures, underscoring the efficacy of integrating reinforcement learning for prediction refinement. A comparison of Trans-D3 with recent hybrid methods reveals that it attains competitive or superior performance, particularly in scenarios characterized by long-term degradation trajectories. In such scenarios, the Transformer’s self-attention mechanism effectively captures temporal dependencies. These results suggest that framing RUL prediction refinement as a control problem provides a complementary advantage over existing data-driven approaches, rather than relying solely on deeper or more complex network architectures.

Notwithstanding the observed performance enhancements, it is imperative to acknowledge the study’s inherent limitations. First, the evaluation is performed exclusively on the CMAPSS benchmark, which, although widely adopted, represents a simulated environment with predefined degradation patterns. Consequently, the reported improvements may not directly translate to industrial systems with different operating regimes or failure modes. Secondly, the efficacy of TD3-based correction is contingent upon the quality of the Transformer’s initial predictions, which may impede resilience under conditions of substantial domain shift. Moreover, the incorporation of a reinforcement learning component contributes to the augmentation of the intricacy of the training process, necessitating meticulous reward design and hyperparameter tuning. Addressing these limitations through cross-domain validation, adaptive reward formulations, and evaluation on real-world datasets is an important direction for future work.

The 50% improvement over supervised-only models indicates that reinforcement acts as a dynamic optimizer that adapts to each evaluated unit rather than as an independent predictor. This suggests that the correction mechanism learns a general policy and develops the ability to adjust predictions based on the error of each motor unit. Such behavior would be challenging to replicate using traditional post-processing methods based on regression or linear filters.

Another relevant finding is that the hybrid model exhibited less variance in the final predictions. In supervised models, error dispersion tends to be greater in engines exhibiting atypical behavior or abrupt degradation paths. However, introducing a control loop using TD3 caused the system to act as a regulator, attenuating oscillations in the error and stabilizing the prediction, even in high-uncertainty scenarios.

One element worthy of discussion is the reward function’s design based on the LQR principle. This formulation was effective because it minimized absolute error and penalized exaggerated corrections that could have undermined the agent’s stability. In contrast to standard reinforcement approaches, where actions can become erratic in the early stages, the function contributed to more progressive learning and a smoother policy.

However, it is important to highlight the limitations related to computational complexity. Although the Transformer offers substantial advantages in feature extraction, its computational cost increases quadratically with sequence length. Although this impact was mitigated by selecting the appropriate sequence length parameter, future research could explore more efficient models, such as Informer or Performer, to reduce training times without sacrificing accuracy.

On the other hand, the training of the TD3 agent critically depends on the quality of the Transformer’s initial predictions. In scenarios where the Transformer makes very large errors, the agent may require a greater number of episodes to converge to a stable policy. This raises the need to study additional pre-training methods or initial error-cleaning pipelines that facilitate reinforcement learning.

It is also pertinent to analyze the model’s ability to generalize across different operating conditions. The analysis suggests that the hybrid architecture maintains its performance even in engines with unusual profiles. However, as it is a system trained with CMAPSS, it would be necessary to validate its robustness in real environments, where noisy sensors, missing values, or simultaneous failures may exist. Adapting the TD3 agent to these contexts may require adjustments to the state definition or reward function.

In terms of interpretability, attention mechanisms made it possible to identify which sensors contribute most to the prediction process. This is in contrast to LSTM or CNN models, where internal relationships are often more difficult to trace. Visualizing attention maps helps us understand how degradation evolves and can be useful for maintenance technicians who need to justify data-driven decisions.

However, the reinforcement component adds an additional level of opacity because the learned policy is not always easily interpretable. Although it improves performance, future research could focus on developing methods to interpret policies or analyze action sensitivity to increase the model’s transparency.

The statistical robustness of the TRANS-D3 model is further evidenced by the high values achieved for the $R^{2}$ and *r* coefficient in all CMAPSS subsets. The model attained an $R^{2}$ of 0.9984 in the FD001 scenario, signifying that the hybrid architecture effectively captures the vast majority of the variability associated with the engine degradation process. This finding persisted even in the most intricate datasets, such as FD002 and FD004, which encompass a multitude of operating conditions and failure modes. Notably, the $R^{2}$ value consistently maintained an above 0.93 threshold, underscoring the model’s efficacy in capturing complex system behavior. Furthermore, the Pearson correlation coefficient (r>0.96) reinforces the linear consistency between the correct predictions and the true RUL, confirming that the model maintains its accuracy throughout the entire operating history of the monitored units.

A critical distinction is made between the uncertainty analysis provided by the 5th and 95th percentiles and the 95% CI for the mean error. The percentiles illustrate the dispersion of individual prediction errors, thereby reflecting the model’s sensitivity to sensor noise and stochasticity. Conversely, the narrow 95% confidence interval for the mean error assesses the statistical certainty of the system bias. For instance, in subset FD004, the confidence interval (CI) of [−1.324, 1.748] ensures that the model is a statistically unbiased estimator. This dual validation approach, which quantifies both the dispersion of individual predictions and systematic bias, provides a transparent and reliable profile of model performance. This profile is a critical requirement for decision-making in safety-critical industrial environments.

The uncertainty analysis underscores a salient strength of the proposed TRANS-D3 framework: its capacity to generate precise predictions and consistent, dependable error behavior under heterogeneous operating conditions. In contrast to conventional deep learning approaches, which primarily optimize point estimate accuracy, TRANS-D3 employs a control-oriented formulation that explicitly restricts prediction corrections. This design decision is reflected in the compact and centered error distributions observed across all CMAPSS subsets, especially in complex scenarios such as FD002 and FD004. The paucity of extreme deviations indicates that the integration of the TD3 agent with an LQR-based reward function effectively mitigates the sudden prediction changes commonly observed in purely supervised architectures when exposed to high operational variability.

Also, the results obtained in the N-CMAPSS DS01 subset indicate that the proposed TRANS-D3 framework effectively balances predictive accuracy and risk-sensitive performance. While the low RMSE demonstrates accurate RUL estimation, the favorable SCORE value suggests that the reinforcement learning-based correction mechanism successfully mitigates critical late prediction errors, which are heavily penalized in forecasting applications. The high $R^{2}$ further confirms that the Transformer–TD3 hybrid architecture captures the underlying degradation patterns. However, it is important to note that this performance comes at the expense of high computational cost, mainly due to the sequential training of the Transformer model and the reinforcement learning agent. This aspect should be carefully considered when implementing the proposed approach in real-time or resource-constrained industrial environments.

From an industrial and Industry 4.0 perspective, these findings carry significant ramifications. Predictive maintenance systems are frequently implemented in safety-critical environments, where decision-making is influenced by more than just the estimated RUL; the confidence associated with that estimate is also a crucial factor. By explicitly characterizing uncertainty using percentile-based error bounds, TRANS-D3 enables maintenance planners to assess risk levels and adopt more conservative strategies when necessary. This distinguishing feature sets the proposed approach apart from existing CMAPSS-based methods, which typically report root mean square error (RMSE) and score without taking prediction reliability into account. Consequently, TRANS-D3 represents a state-of-the-art advancement by bridging the gap between high predictive accuracy and operational reliability, reinforcing its suitability for implementation in complex real-world industrial systems.

Finally, the analysis shows that the Trans-D3 architecture improves not only quantitative performance but also introduces a novel paradigm: the controlled correction of supervised predictions through reinforcement learning. This concept opens up possibilities for new hybrid systems that can be applied to more than just RUL but also to problems such as degradation estimation, maintenance optimization, and composite failure prediction.

Despite the demonstrated robustness of the Trans-D3 architecture in the CMAPSS benchmark, it is imperative to acknowledge the limitations of the proposed implementation in real-world Industry 4.0 environments. First, CMAPSS is a high-fidelity simulated dataset with well-structured sensor measurements and clearly defined degradation trajectories. In contrast, real industrial systems often feature noisy signals, missing data, sensor drift, and unmodeled operational disturbances. Secondly, the efficacy of the correction stage of reinforcement learning is contingent upon the quality of the Transformer’s baseline predictions. Consequently, extreme domain shifts or unforeseen failure mechanisms may necessitate additional adaptation or retraining to ensure the stable convergence of the TD3 agent. Additionally, disparities in sensor availability and configuration across industrial platforms may impede the direct transferability of learned representations. In conclusion, while the proposed reward formulation enhances stability, the reinforcement learning component introduces an additional computational overhead during training, which may restrict its applicability in environments with limited resources.

Notwithstanding these challenges, the Trans-D3 modular design enables adaptation and extension of the domain to real-world predictive maintenance systems. Future research will concentrate on enhancing robustness under real-world sensor conditions, incorporating unlabeled data, and validating the approach in industrial-scale implementations.

## 6. Conclusions

A comparative analysis of the proposed model and traditional approaches shows that incorporating advanced architectures, especially attention mechanisms and contextual dynamics, significantly improves prediction and diagnosis in complex industrial scenarios. The results show increased accuracy, stability, and generalization capacity. This confirms that the proposed approach optimizes the prediction of critical variables and remains robust in the face of real systems’ inherent variability. This robustness is especially apparent in challenging datasets, where conventional methods tend to degrade.

Similarly, the study confirms that the proposed model strikes a favorable balance between computational complexity and performance, which is valuable for applications where inference cost is a relevant factor. Evaluating the model in different operational scenarios validated its adaptability and ability to capture long-range nonlinear relationships. This demonstrates that the proposed architecture is suitable for modern industrial environments where data dynamics are highly variable.

The experimental evaluation at the CMAPSS reference point quantitatively confirms the effectiveness of the proposed TRANS-D3 framework. With regard to prediction accuracy, the model attains considerable RMSE reductions of up to 84–90% under reference operating conditions (FD001) and 23–45% in highly variable and complex environments (FD003 and FD004). Moreover, TRANS-D3 has been demonstrated to enhance the score metric, achieving a reduction in penalty scores of approximately 80–95% in comparison to contemporary SOTA architectures such as STAR and DAST. The numerical results demonstrate that formulating RUL prediction refinement as a control problem and integrating transformer-based temporal modeling with a TD3 agent guided by an LQR-inspired reward function enables highly accurate, stable, and conservative predictions. These predictions are fundamental requirements for predictive maintenance applications in Industry 4.0.

As a future line of research, we propose leveraging the large amount of unlabeled data available in industrial settings to improve the model’s predictive power through semi-supervised or self-supervised learning. Strategies such as temporal consistency, dynamic pseudo-labeling, and pre-trained models on unlabeled sequences could enable the system to learn richer, more generalizable representations. This would reduce dependence on costly and limited fully labeled training sets. Integrating these techniques would pave the way for models that can adapt continuously and efficiently to changing conditions without requiring significant manual annotation.

## Figures and Tables

**Table 1:** Related works to RUL prediction.

Year	Reference	Dataset	Method	Main Advantages	Identified Research Gaps
2021	Mo et al. [32]	CMAPSS	Gated CNN–Transformer	Effectively captures long-term temporal dependencies in degradation trajectories	Fully supervised approach; lacks explicit error correction or control-based mechanism
2023	Ren et al. [33]	CMAPSS	DLformer	Adaptive sequence length improves representation efficiency and computational stability	No reinforcement learning or control-oriented optimization strategy
2023	Li et al. [34]	CMAPSS	CNN-LSTM-SAM	Strong fusion of local and global features through attention-based temporal modeling	Prediction refinement and error correction are not explicitly addressed
2023	Fan et al. [35]	CMAPSS	BiLSTM-DAE-Transformer	Robust feature representation enabled by autoencoder-enhanced bidirectional modeling	Error correction remains implicit and non-adaptive
2024	Liu et al. [36]	CMAPSS	Multiscale transfer learning	Improved RUL prediction performance under limited labeled data conditions	Still purely supervised; lacks feedback-driven or control-based refinement
2025	Dida et al. [37]	CMAPSS	Attention-LSTM	Enhanced temporal focus leading to more stable and accurate predictions	No reinforcement learning or control-theoretic correction mechanism

**Table 2:** Parameters chosen for hyper-optimization.

Parameter	Value
*dim model*	[32, 64, 128]
*num head*	[2, 4, 8]
*num layers*	[1, 4]
*dim feedforward*	[128, 256, 512]
*learning rate*	[$1\times 10^{-5}$, $1\times 10^{-3}$]
*sequence length*	[50, 70, 100]

**Table 3:** Values of the parameters chosen after hyper-optimization.

Parameter	FD001	FD002	FD003	FD004
*dim model*	64	128	64	64
*num head*	2	2	4	8
*num layers*	1	1	4	2
*dim feedforward*	256	512	512	512
*batch size*	64	32	32	32
*learning rate*	0.00014	$9.75\times 10^{-5}$	$8.44\times 10^{-5}$	0.00016
*sequence length*	70	50	70	100

**Table 4:** Hyper-optimized parameters of the TD3.

Parameter	Value	FD001, FD002, FD003, FD004
*labmda penalty*	[0.01, 1]	0.0158
*action low*	[−30, −1]	−28.31
*action high*	[1, 30]	29.995
*learning rate*	[$1\times 10^{-5}$, $1\times 10^{-2}$]	0.000156
*buffer size*	[5000, 10,000]	11,822
*batch size*	[32, 64, 128, 256]	128
*tau*	[0.001, 0.002]	0.0029
*gamma*	[0.9, 0.999]	0.9207
*noise sigma*	[0.1, 1]	0.175

**Table 5:** Summary of the CMAPSS dataset.

Datasets	FD001	FD002	FD003	FD004
**Number of training engines**	100	260	100	249
**Number of test**	100	259	100	248
**Number of operating conditions**	1	6	1	6
**Number of fault modes**	1	1	2	2

**Table 6:** Technical specifications and nomenclature of the turbine engine sensors (C-MAPSS).

Acronym	Physical Parameter	Measurement Unit
T2	Total temperature at the fan section inlet	R
T24	Total temperature at the LPC interface	R
T30	Total temperature at the HPC interface	R
T50	Total temperature at the LPT interface	R
P2	Ambient pressure at the fan intake	psia
P15	Total pressure within the bypass conduit	psia
P30	Total pressure at the HPC discharge	psia
Nf	Rotational speed of the physical fan	rpm
Ne	Rotational speed of the core engine	rpm
epr	Pressure ratio of the engine system	–
Ps30	Static pressure measured at the HPC exit	psia
Phi	Fuel flow rate relative to Ps30 ratio	pps/psi
NRf	Corrected rotational speed of the fan	rpm
NRe	Corrected rotational speed of the core	rpm
BPR	Engine bypass ratio	–
farB	Burner-specific fuel–air ratio	–
htBleed	Enthalpy of the bleed air	–
Bf-dmd	Demand signal for fan speed	rpm
PCNfR-dmd	Demand signal for corrected fan speed	rpm
W31	HPT coolant bleed flow	lbm/s
W32	LPT coolant bleed flow	lbm/s

**Table 7:** Models performance summary.

Model	FD001		FD002		FD003		FD004	
	RMSE	Score	RMSE	Score	RMSE	Score	RMSE	Score
SVR	25.12	4630	27.94	414,000	26.65	15,500	28.13	788,100
MLP	29.15	27,100	33.65	434,000	31.52	27,300	33.13	108,000
CNN	21.51	3980	26.54	28,700	21.36	9650	26.77	8460
LSTM	19.40	1196	20.81	11,900	17.79	5180	27.17	5560
CNN+LSTM	16.12	303	20.45	3440	17.12	1420	23.25	4630
BiLSTM	13.65	295	23.18	4130	13.74	317	24.86	5430
DCNN	12.61	237	22.36	1041	12.64	284	23.31	12,466
GCT	11.27	–	22.81	–	11.42	–	24.86	–
BiLSTM-Att	13.78	255	15.94	1280	14.36	438	16.96	1650
ELSTMNN	18.22	571	–	–	23.21	839	–	–
DATCN	11.78	229	16.95	1842	11.56	257	18.23	2317
AGCNN	12.42	225	19.43	1492	13.39	227	21.50	3392
DAST	11.43	203	15.25	924	11.32	154	18.23	1490
DLformer	–	–	15.93	1283	–	–	15.86	1601
1D-CNN-LSTM	16.10	437.2	–	–	–	–	–	–
CNN-LSTM-SAM	12.60	261	18.90	1156	12.50	253	20.50	2425
BiLSTM-DAE-Transformer	10.98	186	16.12	2937	11.14	252	18.15	3480
STAR	10.61	169	13.47	784	10.71	202	15.87	1449
**Proposed hybrid model (TRANS-D3)**	**1.66**	**8.82**	**13.23**	**114.33**	**6.61**	**42.88**	**12.26**	**406.93**

**Table 8:** Statistical summary and correlation metrics for RUL prediction.

Dataset	Coefficient of Determination ($R^{2}$)	Pearson’s Correlation (*r*)	95% CI for Mean Error
FD001	0.9984	0.9995	[0.709, 1.244]
FD002	0.9339	0.9696	[−1.100, 2.288]
FD003	0.9745	0.9889	[−3.613, −1.154]
FD004	0.9495	0.9745	[−1.324, 1.748]

**Table 9:** Prediction uncertainty statistics across CMAPSS datasets.

Dataset	Mean Error	Std. Dev.	5th Perc.	95th Perc.
FD001	−0.98	1.34	−2.54	0.48
FD002	−0.59	13.82	−23.69	16.47
FD003	2.38	6.17	−3.10	16.13
FD004	−0.21	12.25	−17.46	14.99

**Table 10:** TRANS-D3 performance summary in N-CMAPPS dataset.

Dataset	RMSE	Score	$R^{2}$
DS01	3.57	64.13	0.9624

## Data Availability

The original contributions presented in the study are included in the article; further inquiries can be directed to the corresponding author.
